# Trust in COVID-19 information sources and perceived risk among smokers: A nationally representative survey

**DOI:** 10.1371/journal.pone.0262097

**Published:** 2022-01-27

**Authors:** Reed M. Reynolds, Scott R. Weaver, Amy L. Nyman, Michael P. Eriksen

**Affiliations:** School of Public Health, Georgia State University, Atlanta, Georgia, United States of America; Shahjalal University of Science and Technology, BANGLADESH

## Abstract

**Background:**

Public health officials have classified smoking as a risk factor for COVID-19 disease severity. Smokers generally have less trust in health experts than do nonsmokers, leading to reduced risk perceptions. This study addresses smokers’ trust in information sources about COVID-19 and how trust is associated with perceived COVID-19 susceptibility and severity among smokers.

**Methods and findings:**

A nationally representative sample of 1,223 current smokers were surveyed between October and November 2020, indicating their level of trust in COVID-19 information sources, and their perceptions of risk from COVID-19. Multiple differences in trustworthiness emerged; smokers trusted their personal doctor for information about COVID-19 more than other information sources, while news media were generally distrusted. In addition, the FDA was trusted less than the NIH and CDC. Several “trust gaps” were observed, indicating disparities in levels of trust associated with gender, ethnicity, education, and political orientation, which had the strongest association with trust of all factors. Political orientation was also a significant predictor of COVID-19 risk perceptions, but there was no independent effect of political orientation when accounting for trust, which was predictive of all risk perception outcomes.

**Conclusions:**

Trusted sources, such as personal doctors, may most effectively convey COVID-19 information across political orientations and sociodemographic groups. News media may be ineffective at informing smokers due to their low credibility. The results suggest that trust may explain the apparent effect of political orientation on COVID-19 risk perceptions. Implications for researchers, communication professionals, and policy makers are discussed.

## Introduction

While cigarette smoking’s link to SARS-CoV-2 infection and COVID-19 outcomes remains under investigation [1, 2], national public health officials classified smoking as a risk factor for COVID-19 disease severity, and some jurisdictions prioritized smokers for vaccination, as they are considered a priority population by the U.S. CDC for receiving a COVID-19 booster [3]. Despite these elevated health risks, smokers show disproportionately low willingness to receive the COVID-19 vaccine relative to the general population [4]. The compounding effects of smoking-related illness, COVID-19-related illness, and vaccine hesitancy exacerbate an already dire public health crisis [5]. The ability of policy-makers to promote protective behavior among smokers depends on understanding the bases of smokers’ resistance to such measures. One prominent cause of resistance to public health measures during the COVID-19 pandemic has been mistrust in public institutions and traditional sources for medical information, such as the CDC, FDA, and media organizations [6, 7]. Yet, insufficient research has examined this phenomenon among smokers specifically, although smokers are known to differ from the general population in key respects.

In this study, we examine the extent to which smokers’ trust in COVID-19 information sources may explain their COVID-19 risk perceptions and risk mitigation behaviors. This research extends prior work that has found smokers are less trusting of health experts than are nonsmokers, which partially accounts for their reduced risk perceptions and greater use of nicotine products [8]. Here, we report the first nationally representative study of smokers to address (a) whom smokers trust for information about COVID-19 and (b) how trust is associated with perceived COVID-19 susceptibility and severity. We also explore individual differences that prior research has linked to trust and risk perceptions among the general population, including basic demographic factors as well as political orientation [9]. By identifying the strongest links to trust and risk perceptions among smokers, policy makers can more effectively tailor communication efforts to appeal to sub-populations, depending on their receptivity and need for information. Communication efforts can also leverage the most trusted sources of COVID-19 information among smokers in persuasive campaigns or attempt to repair the reputation of institutions that have lost the public’s trust. In this way, the present study has implications for promoting policies of smoking cessation, social distancing, mask wearing, testing, and vaccination uptake among smokers.

### Trust in health information sources

People seek to be informed about goal-relevant issues in order to adapt to changing environments [10]. Threats in particular elicit strong reactions due to psychological phenomena such as loss aversion [11]. In the context of the COVID-19 pandemic, numerous sources have disseminated information about the disease, its routes of transmission, its health consequences, as well as personal behaviors and social policies that mitigate risks [12, 13]. The effect of received information on subsequent beliefs and behaviors is not fully determined by the content of the information itself. Rather, cues such as source credibility play a substantial role in determining whether the information is accepted and then influences behavior [14]. The concept of source credibility has a long history and can be defined as the perception that a source is trustworthy in a given context in that it (a) possesses correct information (e.g., is competent enough to know the truth) and (b) does not communicate in a deceptive or misleading manner (e.g., only makes claims known to be true).

Trust and credibility are widely considered important factors in risk communication situations, particularly when the issue at hand is new or sufficiently complex that the individual lacks the experience, knowledge, or motivation to directly assess the risks or evaluate the arguments of an important societal issue [15, 16]. In these situations, individuals become more reliant upon the risk assessments and management of experts and their institutions (e.g., industry, regulatory agencies, independent experts, and scientists), where trust serves as a peripheral heuristic cue that operates to reduce the complexity of an individual’s risk-benefit assessment [17].

The COVID-19 pandemic has coincided with a proliferation of sources providing health information and misinformation [18]. These include alternative media and social media platforms whose reach extends across borders and social strata. This proliferation functions, at least in part, to compete with institutional messaging, and research has shown that acceptance of heterodox COVID-19 narratives is associated with distrust of public health institutions and scientists [19].

Within the United States, COVID-related policies and health recommendations have been developed and disseminated by governmental sources like the CDC, NIH, FDA, and so forth, but these efforts have received mixed responses among the public. For example, compliance with public health measures such as movement restrictions, social distancing guidelines, and mask requirements has varied systematically with levels of trust in policy-makers during the COVID-19 pandemic [20–22]. Furthermore, people in the United States have generally shown lower trust in their government’s responses to COVID-19 than those in other developed nations [23], and sub-populations within the US, such as those with less education, and ethnic minorities, show even lower trust in public health institutions for information such as the health consequences of e-cigarettes [8]. As the negative effects of coronavirus continue to mount, there remains a need to study public trust of COVID-19 information sources and its potential to influence beliefs and behaviors pertinent to health promotion.

### Key factors associated with trust

The COVID-19 pandemic has been a politically charged phenomenon, leading to strong associations between political orientation and individual responses to the pandemic. This has manifested as differences between liberals and conservatives in perceptions of risk associated with the disease, skepticism toward government policies addressing the pandemic, engagement in protective behavior, and trust in public health institutions [24, 25]. Although reactions have evolved over time and across a varied political landscape, in general, Americans identifying as more liberal have reported greater trust in public health institutions, greater perceived threat of coronavirus, and stronger adherence to protective behaviors such as physical distancing, mask wearing, and vaccination [26].

The link between trust in COVID-19 information sources and political orientation among smokers has not been rigorously studied. Cigarette smokers differ from the general population in important respects. For example, the population of smokers tends to skew more liberal in the United States [27] and is disproportionately Caucasian [28]. Smoking increases risks of severe COVID-19 disease, yet, smokers tend to be more sensation-seeking and risk tolerant [29]. Smokers also tend to have optimistic bias [30] and believe they are unlikely to face consequences of their smoking behavior. Recently, research has shown that persistent smokers with low desire to quit are less likely to consider themselves at risk of severe COVID-19 infection [31]. So, it remains important to observe levels of trust among smokers, and the link between trust and risk perceptions in the context of COVID-19.

### Links between trust, risk perceptions, and behavior

Risk management and communication scholars have noted the critical importance of public trust for understanding people’s attitudes on societal issues with implications for health and safety [32–40]. Studies have found robust associations between trust in risk-communication sources and individual risk perceptions for a multitude of issues relevant to public health [38, 41], including climate change [33], food safety [42], pesticides [37], hazardous waste disposal [43], and artificial sweeteners [37].

Complex and evolving, the COVID-19 pandemic has prompted individuals to make crucial health decisions while, in many cases, lacking the experience and expertise to evaluate the emerging evidence to make informed decisions. Therefore, according to dual-process theories of persuasion [15, 44] these individuals must rely largely on a peripheral information-processing route in forming their opinions about the risk-benefits of policies and behaviors alike. The degree of public trust in sources for information about COVID-19 should play an important role in individual reactions to the conflicting risk communications about COVID-19 that are currently widespread.

Since the onset of the COVID-19 pandemic, emerging research has aimed to further elucidate the associations between trust and risk perceptions in the context of the COVID-19 pandemic [45]. For instance, a multinational study of risk perceptions found that trust in government was negatively associated whereas trust in science and medical professionals were positively associated with risk perceptions [46]. Distinguishing among types of trust, a study conducted in Swiss adults found lower social trust (tendency to trust institutions with perceived similarity in values) but higher general trust (tendency to trust strangers) was associated with high perceived risk of COVID-19 [7]. A limited corpus of research has further linked COVID-19 risk perceptions to attitudes towards government policies to control the spread of COVID-19 and to individual risk-mitigation behaviors [45, 47–49]. For instance, the aforementioned study additionally found and that perceived risks were associated with COVID-19 social distancing behaviors and acceptance of government policies to close schools, restaurants, bars, and shops [7]. In another study, an online survey of a convenience sample found trust in science and COVID-19 risk perceptions were uniquely associated in COVID-19 prevention behaviors and the association between political orientation and behavior may be mediated by trust in science [50]. Despite voluminous literature, there has been limited research examining the association between source trust for COVID-19 information and risk perceptions among the population of smokers who are burdened with elevated risk of COVID-19 morbidity and mortality, and whose level of trust may not resemble the general public. The present study, therefore, aims to measure smokers’ trust in COVID-19 information sources and describe the demographic factors associated with differences in trust levels. In addition, this study examines predictors of COVID-19 risk perceptions, including trust.

## Methods

### Participants

Data were collected through the 2020 (October-November) Tobacco and COVID-19 Survey of a national probability sample drawn from Ipsos Public Affairs’ KnowledgePanel, a probability-based web panel designed to be representative of non-institutionalized U.S. adults [51]. Data collected by KnowledgePanel have been used by numerous national health and research organizations [52–54]. Computers with internet access were provided for panelists who did not have them. Adult panelists (18+ years) who had reported current cigarette smoking or current ENDS use on recent Ipsos’ profile surveys were randomly sampled and invited to participate upon confirmation that they were current users of cigarettes (defined as having smoked at least 100 cigarettes in their lifetime and now smoking “every day” or “some days”) or ENDS (defined as now using ENDS “every day” or “some days”), or had recently (since February 2020) quit smoking cigarettes or using ENDS.

Overall, 2,752 KnowledgePanel members were invited to participate in the survey, of which 1,630 (59.2%) completed the screener survey. Of the 1,535 qualified screener completers, nine were excluded for completing the survey in less than one-third of the median duration time, resulting in a final sample of 1,526 cases. Of these, 1,223 reported current cigarette smoking (the present sample). A final stage completion rate (completed surveys out of total invited) of 55.5% and a qualification rate of 94.2% were obtained. The average panel recruitment rate for this study, reported by Ipsos, was 11.3% and the average profile rate was 62.4%, for a cumulative response rate (the product of panel recruitment rate, profile rate, and screener completion rate) of 4.2% [55]. A study-specific post-stratification weight was computed using an iterative proportional fitting (raking) procedure using benchmarks obtained from the 2019 National Health Interview Survey data (gender, race/ethnicity, census region, metropolitan status, education) and KnowledgePanel profile data (household income). The GSU IRB approved the study as exempt and authorized a waiver for obtaining study-specific consent. Ipsos obtains blanket informed consent from all panelists electronically when they join the panel.

### Materials and measures

#### Trust

To indicate trust, participants answered the question, “how much do you trust what each of the following say about coronavirus?” for the CDC, FDA, NIH, “health experts and scientists”, their “doctor or other medical provider”, and news media. Trust was indicated on a five-point scale ranging from “strongly distrust” (-2) to “strongly trust” (2). All trust items had less than 4% of participants fail to respond or indicate they did not know. Across all trust items, approximately 2.2% of responses were missing or indicated the participant did not know (165 out of 7338).

#### Perceived COVID-19 risk

Participants answered four questions about their perceived COVID-19 risk. First, in terms of *susceptibility* (“How likely do you think you are to be infected by the coronavirus over the next year?”), they responded on a 5-point scale from “unlikely” (1) to “certain” (5). Second, in terms of *severity* (“how severe do you think your symptoms will be if you become infected with coronavirus?”), they responded on an 11-point scale from “I would likely have no symptoms” (0) to “I would likely die from it” (10). This variable was linearly transformed into a 1–5 scale to help standardize the univariate interpretation. Specifically, each score was multiplied by .4 and added to the quantity 1. Using constants to linearly transform the scores fully preserves the distribution of the variable and has no effect on its correlation with other variables. Third, participants indicated how much they believed that smoking cigarettes causes greater COVID-19 susceptibility (“Based on what you believe, how much do you agree or disagree with the following statements? Smoking cigarettes can cause me to be more likely to get coronavirus.” Participants responded on a 5-point scale that ranged from “strongly disagree” (1) to “strongly agree” (5). Fourth, participants indicated how much they believed that smoking cigarettes causes greater COVID-19 severity (“Based on what you believe, how much do you agree or disagree with the following statements? Smoking cigarettes can cause me to have more severe effects of coronavirus.” Participants responded on a 5-point scale that ranged from “strongly disagree” (1) to “strongly agree” (5). For all risk perception variables, less than 7% of participants had a missing a response or indicated they did not know. Across all risk items, approximately 3.6% of responses were missing or indicated the participant did not know (175 out of 4892).

#### Political orientation

Participants indicated their political orientation by responding to the question, “Which of the following best describes how you think of yourself.” Participants responded on a seven-point scale from “extremely liberal” (1) to “extremely conservative” (7), with the midpoint (4) labelled “moderate.” For a more parsimonious and interpretable analysis, we recoded political orientation into three categories: Liberal, Moderate, and Conservative. For this variable, less than 2% of participants were missing a response or indicated they did not know (23 out of 1223).

#### Covariates

**Fig 1:**
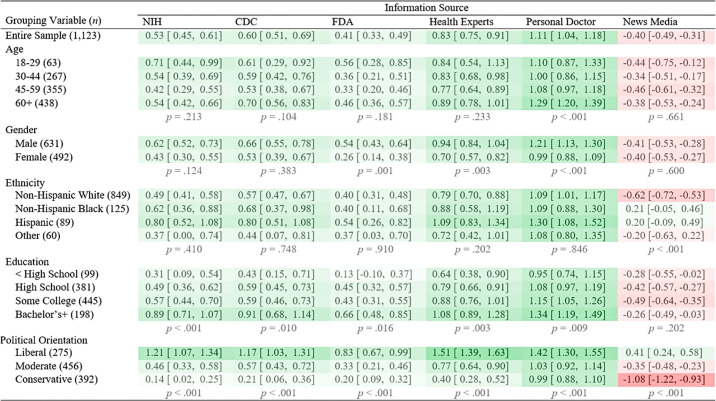
Trust in COVID-19 information sources among smokers (weighted means and CIs). Brackets contain 95% CIs. Sampling probability weights were used. Trust variables used a 5-point scale ranging from “strongly distrust” (-2) to “strongly trust” (2). Darker green corresponds with greater trust; darker red corresponds with greater distrust. Respondents who selected “don’t know” or refused to answer (2.2% of total responses) were excluded from this analysis (listwise). *p* values indicate the significance level of the omnibus test of mean differences for each grouping variable.

Participants indicated their age, gender, ethnicity, education-level, income, marital status, employment status, COVID-19 infection status, family-member COVID-19 infection status, and perceived physical health. Each of these were coded as categorical variables for use as covariates in regression models and for between-group mean comparisons (see Fig 1).

### Analysis

For the analyses, survey-weighted means, 95% CIs, and survey-weighted ordered logistic regressions were estimated using Stata, version 16 (StataCorp LLC). A threshold of *p* < .05 (2-sided) was used for statistical significance tests. Use of survey-weights accounts for differential rates of participation among demographically identifiable sub-populations [56]. The process for generating these weights is described above alongside the sampling procedures. For the regression models, trust ratings of the five health sources (CDC, FDA, NIH, health experts and scientists, personal doctor or medical provider) were averaged into a single scale that showed good reliability (α = .88) and unidimensionality (*CFI* = .979, *SRMR* = .024; see Reynolds [57]). For each analysis, listwise deletion was used to preserve a more consistent analytic sample.

## Results

Sample participants were 44.6% women, 75.2% Non-Hispanic White, 11.3% Non-Hispanic Black, 8.2% Hispanic, and 5.3% other. The mean age was 53.3 years (*SD* = 14.1, *min*. = 18, *max*. = 87). On average, smokers trusted their personal doctor for information about COVID-19 more than other information sources (Fig 1). Health experts and federal agency health sources (NIH, CDC, FDA) were generally trusted; the average smoker was more trusting than distrusting of these sources, notwithstanding variation between these sources. In general, the FDA was less trusted than the CDC, *t*(1165) = 5.59, *p* < .001, and less trusted than the NIH, *t*(1165) = 4.03, *p* < .001 (Survey-weighted t-tests were used). News media were generally distrusted as sources of COVID-19 information.

Political orientation had the strongest association with trust of all factors among smokers. As a group, liberals were the most trusting, followed by moderates, and then conservatives. On average, conservatives were still, albeit slightly, more trusting than distrusting of health sources for COVID-19 information. Only liberals trusted news media (on average) while moderates were distrustful, and conservatives even more so.

Female smokers were generally less trusting in health sources than male smokers, and this effect was most pronounced for trust in the FDA, Health Experts and Scientists, one’s Personal Doctor. Compared to minority smokers, Non-Hispanic White smokers indicated more distrust in news media, but ethnicity was not associated with trust in other information sources. More education was associated with greater trust for every source but news media among smokers, and individuals with at least a bachelor’s degree appeared to be particularly trusting of official health sources and their personal doctor.

Greater trust was associated with greater perceived risk of COVID-19 susceptibility and severity, controlling for other factors (Table 1). Smokers with more trust in health sources were also more likely to believe that smoking increases their COVID-19 susceptibility and severity (if infected). Both trust in health sources as well as trust in news media were independently associated with perceived COVID-19 severity. Although political orientation was a significant correlate of multiple risk perception outcomes (models U), when statistically accounting for trust (models T) there was no independent effect of political orientation.

**Table 1:** Predictors of COVID-19 risk perceptions among smokers (ordered logistic regression).

	Outcome							
	Perceived COVID Susceptibility		Perceived COVID Severity		Belief that Smoking Increases COVID Susceptibility		Belief that Smoking Increases COVID Severity	
Predictor Variable	Model 1U	Model 1T	Model 2U	Model 2T	Model 3U	Model 3T	Model 4U	Model 4T
Political Orientation								
Liberal	1.61^A^[1.03, 2.52]	1.30 [0.84, 2.02]	1.58^A^[1.11, 2.26]	1.03 [0.73, 1.45]	1.39 [0.96, 2.00]	1.14 [0.78, 1.68]	1.68^B^[1.17, 2.41]	1.22 [0.84, 1.76]
Conservative	1.15 [0.80, 1.65]	1.41 [0.94, 2.13]	0.69^A^[0.51, 0.94]	0.94 [0.68, 1.29]	0.74 [0.54, 1.02]	0.85 [0.61, 1.18]	0.69^A^[0.50, 0.95]	0.84 [0.61, 1.17]
Trust (health sources)	-	1.21 [0.95, 1.53]	-	1.57^C^[1.30, 1.91]	-	1.24^A^[1.02, 1.51]	-	1.83^C^[1.49, 2.26]
Trust (news sources)	-	1.23^A^[1.04, 1.45]	-	1.32^C^[1.15, 1.52]	-	1.12 [0.96, 1.31]	-	1.02 [0.89, 1.18]
Age								
18–29	Reference	Reference	Reference	Reference	Reference	Reference	Reference	Reference
30–44	0.86 [0.44, 1.71]	0.86 [0.43, 1.72]	1.56 [0.93, 2.63]	1.61 [0.96, 2.71]	0.95 [0.54, 1.65]	0.93 [0.53, 1.64]	1.07 [0.64, 1.78]	1.07 [0.65, 1.77]
45–59	0.59 [0.30, 1.18]	0.58 [0.29, 1.15]	1.62 [0.97, 2.72]	1.66 [0.98, 2.80]	0.76 [0.42, 1.02]	0.73 [0.40, 1.33]	0.65 [0.38, 1.11]	0.64 [0.37, 1.09]
60+	0.66 [0.31, 1.40]	0.61 [0.29, 1.31]	2.40^B^[1.31, 4.39]	2.18^A^[1.20, 3.99]	0.53 [0.28, 1.02]	0.49^A^[0.25, 0.96]	0.60 [0.33, 1.08]	0.55^A^[0.30, 0.99]
Gender								
Male	Reference	Reference	Reference	Reference	Reference	Reference	Reference	Reference
Female	1.21 [0.90, 1.64]	1.28 [0.94, 1.75]	1.12 [0.85, 1.49]	1.24 [0.94, 1.64]	0.92 [0.70, 1.21]	0.95 [0.72 1.26]	0.83 [0.63, 1.09]	0.90 [0.69, 1.19]
Ethnicity								
Non-Hisp. White	Reference	Reference	Reference	Reference	Reference	Reference	Reference	Reference
Non-Hisp. Black	0.70 [0.42, 1.17]	0.64 [0.39, 1.06]	0.86 [0.54, 1.38]	0.81 [0.51, 1.29]	1.10 [0.68, 1.77]	1.05 [0.65, 1.69]	0.71 [0.45, 1.12]	0.73 [0.46, 1.16]
Hispanic	0.91 [0.47, 1.74]	0.77 [0.17, 1.26]	1.67 [0.93, 2.93]	1.34 [0.75, 1.42]	1.12 [0.59, 2.14]	1.00 [0.66, 2.75]	0.81 [0.44, 1.49]	0.73 [0.40, 1.35]
Other	0.48 [0.18, 1.25]	0.47 [0.40, 1.47]	0.81 [0.45, 1.45]	0.73 [0.44, 1.22]	1.36 [0.68, 2.72]	1.35 [0.65, 1.69]	0.79 [0.47, 1.33]	0.85 [0.48, 1.52]
Education								
< High School	Reference	Reference	Reference	Reference	Reference	Reference	Reference	Reference
High School	0.98 [0.53, 1.83]	0.92 [0.48, 1.73]	1.39 [0.78, 2.45]	1.18 [0.68, 2.07]	2.15^C^[1.37, 3.38]	2.03^B^[1.28, 2.22]	2.51^C^[1.55, 4.07]	2.28^C^[1.40, 3.70]
Some College	1.15 [0.60, 2.23]	1.12 [0.58, 2.16]	1.20 [0.66, 2.18]	1.10 [0.61, 1.98]	1.76^B^[1.11, 2.79]	1.72^A^[1.07, 2.76]	2.32^C^[1.42, 3.80]	2.13^B^[1.27, 3.52]
Bachelor’s+	1.71 [0.73, 4.01]	1.51 [0.64, 3.60]	1.28 [0.64, 2.55]	0.98 [0.50, 1.96]	3.39^C^[1.79, 6.40]	3.11^C^[1.63, 5.96]	3.39^C^[1.86, 6.21]	2.85^C^[1.54, 5.28]
N	1,163		1,159		1,093		1,097	
Mean [95% CI]	1.95 [1.89, 2.02]		3.06 [2.99, 3.13]		2.72 [2.64, 2.80]		3.34 [3.26, 3.42]	

*Note*. Displayed model-coefficients are adjusted odds ratios with 95% CIs within brackets.

^**A**^*p* < .05,

^**B**^*p* < .01,

^**C**^*p* < .001.

For Political Orientation, “Moderate” is the reference category. Models were estimated using survey weights. Risk perception variables were scaled from 1 to 5 with higher scores indicating greater perceived susceptibility/severity. For each outcome, models are reported both with (T) and without (U) the trust predictors. The following adjustment variables were included in addition to those displayed: marital status, status, self-reported physical health, personal and family COVID-19 status. Responses of “don’t know” were excluded.

The models in Table 1 also revealed that additional demographic factors were associated with risk perceptions after controlling for trust. Specifically, more educated smokers were more likely to believe that smoking increases the susceptibility and severity of COVID-19 disease, and this effect appears to be independent of trust. Smokers above the age of 60 were also likely to perceive greater risk from COVID-19 but conversely, those older smokers were less likely to perceive smoking as a contributing factor to their susceptibility to COVID-19 or the severity of COVID-19’s effects.

## Discussion

As the COVID-19 pandemic continues to harm the health and livelihoods of people in the U.S. and worldwide, the health-behaviors enacted by the public, such as vaccination, remain important determinants of the course of the pandemic [58]. The well-established link between risk perceptions and health behavior [59] implies the need to understand the predictors of risk perceptions toward COVID-19, which include trust in COVID-19 information sources. The issue of trust itself then raises questions about the best routes to disseminate critical information, and the need to enhance or rebuild credibility. This study addresses these questions for the population of smokers, which has been understudied in this context, yet remains at elevated risk from COVID-19 and disease in general [1]. The present results show differences of trust levels in major information sources including the NIH, CDC, FDA, health experts and scientists, personal doctors, and news media, as well as demographic factors associated with trust. The present results also demonstrate three important facts about the role of political polarization in the COVID-19 pandemic among smokers: (a) that highly divergent perspectives have emerged along ideological lines about which sources are trustworthy for medical information, (b) that political orientation is associated with perceived risks of COVID-19, but (c) trust is the key variable that explains the apparent effect of polarization on COVID-19 risk perceptions.

Among smokers, distrust of news media’s reporting about COVID-19 was prevalent. Of all identified sub-groups, only politically-liberal smokers were significantly more trusting than distrusting of news media. Political moderates tended to distrust news media about COVID-19 information, while conservatives were profoundly distrustful. Although the extent and nature of bias among news media outlets remains controversial [60, 61], conservatives perceive media bias [62] which undermines effective communication of even non-political issues. Because of this, communication through alternative channels may enhance communication efforts, particularly when conservative audiences are the intended recipient. Personal doctors may partially fulfil this role, as they were the most trusted among smokers, including conservatives (by far). However, an ideological the trust gap still exists for personal doctors, who were less trusted by moderates and conservatives than by liberals. Nonetheless, personal healthcare providers may be the most effective messengers of COVID-19 information across sociodemographic groups and political orientations. These findings also call for additional research to understand the factors that contribute to doctors’ ability to maintain trust with patients. For example, Gopichandran and Chetlapalli [63] identified several “soft skills” that are primary drivers of patient trust. These include establishing a comfortable environment for self-disclosure, developing personal involvement with the patient, and cultural competence.

Gender was also significantly associated with trust in personal doctors, health experts and scientists, and the FDA. For these three sources, women who smoke were less trusting than men who smoke. Although the present results cannot attribute this difference to a particular cause, this raises concern over potential ways in which these sources fail to garner equal trust from women as they do men. For doctor-patient relationships specifically, research has shown that men and women develop trust in response to different cues [64], and doctors may not be adapting sufficiently to the needs of female patients. In addition, doctors have historically been disproportionately male (although trends are reversing [65]), and the gender disparity may contribute to the trust deficit between male and female smokers for doctors. Unequal gender representation may play a role in differences in trust for health experts and the FDA, although future research is needed to confidently identify the factors responsible.

Education was also associated with multiple trust variables. Smokers with more education, and in particular those with at least a bachelor’s degree, were more trusting in all sources except news media. This is consistent with prior findings that education is associated with greater trust in expertise and official sources [8]. This effect may be due to a difference in ability to process information between more and less educated individuals. Alternatively, this could reflect a difference in the value placed on formal training and expertise. Although equal representation of all education levels within critical professions may not be feasible, future research should develop messaging strategies to reduce the trust gap associated with education.

Ethnicity was not generally associated with trust, except in the case of news media. There, minority smokers did not significantly differ from one another, but Caucasian smokers were significantly distrusting of news media, and significantly less trusting than each minority group. Although the cause of this trust gap remains uncertain, it is worth considering possible sources of influence. In the US, mass media has traditionally under-represented minority groups or portrayed minorities according to inaccurate stereotypes [66]. Although disparities in representation persevere in media, the prevalence of Caucasian-centric content has declined somewhat, the inclusion of diverse cast members has increased, and insensitive stereotypes of ethnic minorities appear less frequently [67]. While none of this implies an anti-Caucasian bias, the drift away from a pro-Caucasian bias may disrupt the relationship many once had with media content. Contemporary events must also be considered, as data for the present study were collected mere months after the murder of George Floyd and prolonged media coverage of racially charged protests and civil unrest [67] that drew attention to police brutality [68] but also exacerbated tensions along racial and political lines [69]. This may partially explain the significant distrust of news media among Caucasian smokers if they felt reactance against this coverage. Because credibility accrues to a source over time it may not be context-specific, and perceived violations of trust may spill over into other contexts involving that source.

In addition to utilizing more trusted sources to disseminate information, the present results show a need to repair credibility, particularly of news media, News media were the least trustworthy of all measured sources, and especially to political conservatives. Improving trust may therefore be difficult for that population, however, there is also more opportunity to make gains. More research should investigate the causes of conservatives’ distrust of news media beyond surface-level perceptions of bias. Message exposure experiments should be conducted to identify the kinds of messaging that are most and least appealing to conservative smokers. For example, research can test the effectiveness of acknowledging values generally held by conservatives, regardless of the specific policy recommendation. Health recommendations may be couched in terms of conservative values, for example by emphasizing that compliance leads to a hastened end to government restrictions. For this purpose, researchers and message designers can draw from the Moral Foundations Theory literature [70]. Simply cultivating basic elements of credibility may be effective broadly. This involves sustained demonstration of competence, goodwill, and integrity in the eyes of skeptical sub-populations. Importantly, repairing trust is complicated by the tendency of mistrust to arouse skepticism of attempts to build trust. Sometimes the reputation of a firm, an agency, or an individual cannot be salvaged [71], and new entities must be created.

This study showed that, for smokers, trust plays a consistent role in COVID-19 risk perceptions, which are a critical predictor of risk-mitigating behaviors [59, 72]. Smokers who were more trusting of official health sources were more likely to perceive the effects of COVID-19 as severe and were more likely to believe smoking increases their susceptibility to COVID-19 and the severity of COVID-19, should they become infected. Smokers who were more trusting off news media perceived themselves as more susceptible to infection. The present results provide cross-sectional data consistent with trust as a mediator of the link between political orientation and risk perceptions. Although political orientation was correlated with COVID-19 risk perceptions, it had no significant effect on any measured risk perception when controlling for trust. This highlights the importance of trust as a plausible mechanism by which political orientation may influence health-promoting behavior in a politically charged context.

Political action often involves competition among groups for scarce resources [73], leading to conflict. In a state of conflict, honest communication is potentially detrimental if it provides the adversary with useful information. Conversely, dishonest communication is an opportunity to manipulate. For these reasons, excessive politicization of issues, such as health recommendations, may generally erode trust between social groups. Perversely, however, it may benefit in-group cohesion. The problem of mistrust and misinformation is complex and perhaps intractable, but whatever solution may emerge will need to address the incentives that perpetuate misinformation and mistrust. Some have proposed more stringent regulations against misinformation in media [74], while others emphasize the drawbacks of censorship [75]. Despite the difficulty in arriving at clear policy recommendations, the need for additional research is obvious.

The link between trust and risk perceptions is at least partially consistent with dual-process theories of persuasion [15, 44]. According to these theories, source credibility should be particularly impactful when the ability to understand or validate information is low. This has been the case during the COVID-19 pandemic in several respects. For example, the novelty of the virus increases the difficulty of using prior knowledge to predict likely outcomes. In addition, the effectiveness of health-measures is not directly observable by members of public but are measured across populations, locations, and with sophisticated instruments. In situations of this type people are less persuaded by arguments themselves, owing to the difficulty of evaluating the evidence, but rather they rely on sources they deem trustworthy. This explains the significant association between trust and risk perceptions because the evidence is not able to speak for itself, and people following different sources can easily come to different conclusions. On the other hand, the pandemic has been so impactful, people are motivated to seek and process information about it (i.e., “do their own research”) [76]. Nonetheless, motivation cannot overcome a deficit in ability to process information, unless that ability is enhanced.

Education was also significantly associated with risk perceptions, controlling for all other predictors. Specifically, more educated smokers (especially college graduates) were more likely to believe that smoking cigarettes increased their susceptibility to COVID-19 infection, and that the severity of the disease would be greater, should they be infected. In contrast, education was not associated with perceived COVID-19 severity nor with their perceived personal susceptibility to infection. This effect may be a result of multiple processes. For example, through selective exposure, more educated individuals may have been more likely to encounter news stories or have discussions about cigarettes’ ability to increase severity of COVID-19 infection. Future research should investigate the cause of these differences. Regardless of the mechanism, this result suggests that the COVID-19 pandemic has persuasive potential. People who strongly believe that cigarettes make them more vulnerable to COVID-19 may be more motivated to quit smoking. There may be benefit in targeting more educated smokers with smoking cessation messages and resources that reference the unique risks of smoking combined with COVID-19. On the other hand, recent research suggests that the COVID-19 pandemic has led some smokers to consume more cigarettes, despite awareness of the health effects [77]. This is partly due to increased stress, for which smoking is a coping mechanism. Therefore, it is important to consider backfire effects with any intervention.

### Limitations

The findings of this study should be considered alongside multiple limitations. First, the data from this study are a cross-sectional sample collected at a single point in time. The pandemic has evolved and had many stages corresponding to changes in case-rates, health-measures, vaccine availability, and so forth. The results presented here are a valuable snapshot, but research should continue to investigate trust in COVID-19 information sources among smokers and their association with risk perceptions. Longitudinal designs would enable greater confidence about the causal process and allow description of trends over time as the situation changes.

This study shows the importance of trust for risk perceptions, which are known to predict behavior, however, in this study we did not observe behavior directly. Doing so could help test the health belief model [59] in this context to determine which risk perceptions are most predictive of smoking cessation or other health-behavior changes. Additional variable such as efficacy could be added to determine the extent to which smokers feel able to control their health, either through actions directed toward COVID-19 or through reduction in smoking.

This study was able to measure trust in several COVID-19 health sources, yet, future research could benefit from an even more extensive set of trust variables. For example, more nuanced indicators can assess trust in specific public figures or media personalities. This is relevant because news media are a heterogenous collection of programs. It could be meaningful to detect clusters of individuals whose trust of particular segments of media may differ from their trust of media overall. The same case can be made regarding social media, which encompasses numerous platforms, each of which are populated by user-driven content, community interaction, and innumerable content creators. Because of social media’s individualized nature, users on the same platform may be exposed to completely difference messages. These challenges should continue to be addressed.
